# The relation between dietary phytochemical index and metabolic syndrome and its components in a large sample of Iranian adults: a population-based study

**DOI:** 10.1186/s12889-021-11590-2

**Published:** 2021-08-24

**Authors:** Azam Ahmadi Vasmehjani, Zahra Darabi, Azadeh Nadjarzadeh, Masoud Mirzaei, Mahdieh Hosseinzadeh

**Affiliations:** 1 Nutrition and Food Security Research Center, School of Public Health, Shahid Sadoughi University of Medical Sciences, Yazd, Iran; 2 Department of Nutrition, School of Public Health, Shahid Sadughi University of Medical Sciences, Yazd, Iran; 3 Yazd Cardiovascular Research Center, Shahid Sadoughi University of Medical Sciences, Yazd, Iran

**Keywords:** Dietary phytochemical index, Metabolic syndrome, Phytochemical-rich foods, Triglyceride, Hypertension, Iran

## Abstract

**Background:**

Despite the protective effects of foods being rich in phytochemicals against chronic diseases, this issue is still poorly understood. The aim of this study was to investigate the association between Dietary Phytochemical Index (DPI) and metabolic syndrome (MetS) and its components.

**Methods:**

This cross-sectional study focused on adults aged between 20 and 70years**.** The dietary intake was assessed using a validated and reliable food frequency questionnaire. DPI was calculated based on dietary energy, derived from phytochemical-rich food sources (kcal) per total daily energy intake (kcal). The odds ratio of MetS and its components were assessed across DPI quartiles by logistic regression models**.**

**Results:**

After adjustment for all potential confounders, the risk of MetS (OR: 0.63, 95% CI = 0.41–0.96) and elevated blood pressure (OR: 0.62, 95% CI = 0.40–0.96) in the second category of DPI decreased significantly as compared to that in the first category. Subjects in the second and fourth quartiles of DPI with adjusting for age, sex and total energy intake revealed 30 and 25% lower risk of abdominal obesity, respectively. After full adjustment for confounders, the analysis stratified by sex showed women in the highest quartile of DPI had 59% lower risk of MetS (OR: 0.41, 95% CI = 0.22–0.76) as compared to those in the lowest quartile of DPI.

**Conclusions:**

Greater adherence to phytochemical-rich diet could reduce odds of MetS and some components, especially in women. Further studies with intervention approaches are recommended.

## Background

A set of cardiovascular risk factors including abdominal obesity, hypertension, dyslipidemia, glucose intolerance, and insulin resistance indicates Metabolic Syndrome (MetS) [1, 2]. The global prevalence of MetS has been reported from smaller than 10 to 85% [3]. Recent national data showed that more than 30% of Iranian adults suffer MetS [4]. Various factors including race, family history of diabetes, hypertension and heart disease, genetics, age, gender, lifestyle, diet and obesity are involved in the development of MetS [5, 6]. Identification of the modifiable factors such as diet is essential to prevent the development of MetS [7].

Former studies have demonstrated that increased levels of saturated fat and cholesterol [8], animal protein [9, 10], and high glycemic index diet [11] lead to the risk of MetS. In contrast, higher intake of unsaturated fat [8], fruit and vegetables [12] help reduce the risk of MetS.

Longstanding studies on the aforementioned issue have focused on nutrients, foods, and food groups, with less emphasis on dietary patterns. Since nutrient interactions cannot fully explain the association between food and chronic disease, dietary patterns have been proposed as a new approach in nutritional studies [13, 14]. Recently, nutritionists have focused on the combined effects of food, proposing a multivariate approach of food patterns [15]. The findings of these studies have proven that the Mediterranean dietary pattern which is rich in vegetables and fruit, nuts, legumes, olive oil; low in saturated fat, red meat and poultry; moderate in fish; and low to medium in dairy products yield beneficial health effects through phytochemicals [16]. Phytochemicals are natural non-nutritive bioactive compounds including phenolic, isoperenoids and organosulfor compounds [17, 18]. Due to the health-boosting effects of phytochemicals, dietary phytochemical index (DPI) was suggested by McCarty, which is defined as a percentage of calories derived from food rich in phytochemicals [17]. DPI calculation seems to be a simple and inexpensive method of assessing the background of dietary quality as well as clinical applications [19].

So far, some studies have examined the relationship between DPI and health indicators including oxidative stress, inflammation, cancer and mental health [20–22**].** Prior findings have shown that an affluent diet in phytochemicals via antioxidant and anti-inflammatory properties plays a protective role in the development of insulin resistance, abnormal glucose, lipid disturbances and abdominal obesity [23–26]. However, the relation between DPI and the occurrence of MetS is still poorly understood. As such, the purpose of this study was to investigate the association between DPI and the risk of developing MetS and its components in a large population of Iranian adults.

## Methods

### Study design and participants

This cross-sectional study was carried out on data obtained from recruitment phase of Yazd Health Study (YaHS) and Taghzieh Mardom-e-YaZd (TAMYZ) conducted from 2014 to 2016. YaHS is a population-based prospective cohort study of 10,000 people aged 20–70 years who randomly selected from 200 clusters (50 each, 25 men and 25 women and 5 persons in each ten years age groups) of Yazd greater area according to the city post codes. TAMYZ was a nutrition sub-study of YaHS which was conducted on the same 10,000 participants, however only 8000 of them were participated. Over 100 trained interviewers visited the participants at their residence after set up meeting time and then filled a validated questionnaire with 300 questions including dietary intakes demographics and physical activity. All participants were invited to attend a referral laboratory within two weeks from the interview date to provide fasting blood sample for biochemical assessments. The profile of the studies was published elsewhere [27].

**Fig. 1:**
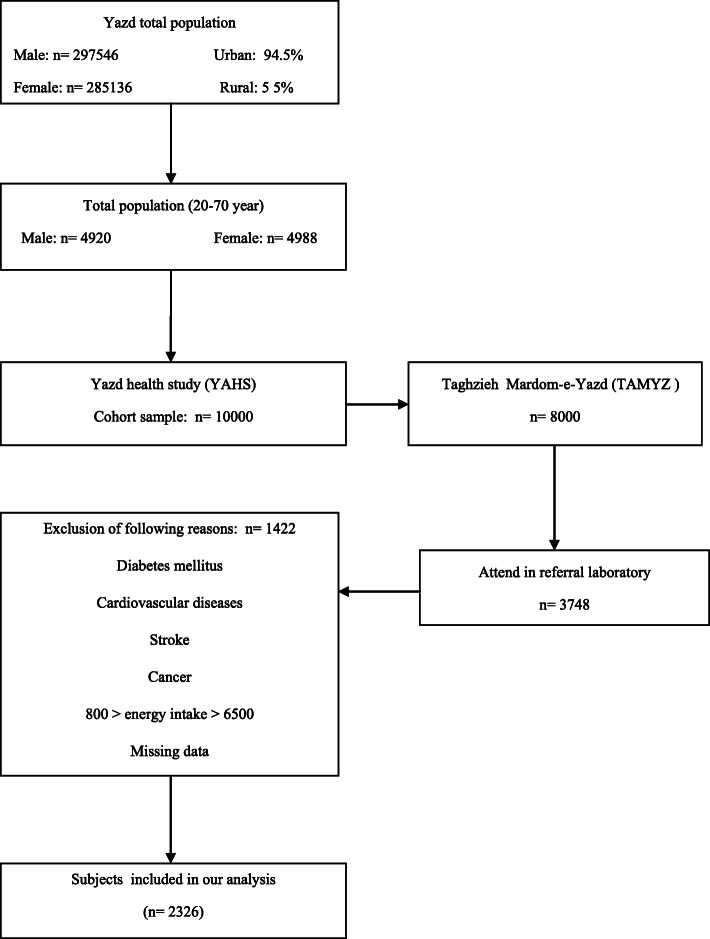
Flowchart of the data collection process of study

Almost 40% of the YaHs participants gave consent for fasting blood sampling and biochemical assessment; there was no significant difference between those who attends and those who do not attend in terms of major SES factors. Out of 3748 available cases with data on dietary intakes, blood tests and main variables associated with MetS, the subjects with following conditions were excluded: having history of diseases such as diabetes mellitus, cardiovascular diseases, stroke and cancer or persons whose total daily energy intake was less than 800 or higher than 6500 kcal and missing data. Finally, 2326 subjects were included in this analysis. Flowchart of the data collection process is shown in Fig. 1.

Written informed consent was obtained from all participants. Ethical approval was obtained from the Ethics Committee of Shahid Sadoughi University of Medical Sciences, IR.SSU.SPH.REC.1399.051. YaHS database is not publically available. The authors assess the data according to the study protocol and agreement with the CI of study Professor Masoud Mirzaei who critically read and comment on this manuscript.

### Dietary assessment

Dietary intakes were assessed through a validated (FFQ) consisting of 178 food items which was modified version of a previously validated 168-item FFQ. Additional 10 questions relating to consumption of Yazd-specific food items were added to the original FFQ that were collected by trained interviewers [27, 28]. Participants were asked about the frequency and usual amount consumption of food items in the past year then were converted to grams using guidelines of household scales [29].

### Phytochemical index calculation

The DPI was calculated based on the method developed by McCarty in 2004; [[DPI = (daily energy derived from phytochemical-rich foods (kcal)/total daily energy intake (kcal)) × 100) [17]. Fruits, vegetables, legumes, whole grains, nuts, soy products, seeds and extra virgin olive oil were considered as phytochemical-rich foods. Coffee and tea, as free energy sources, were not contributed. Potatoes were not included as vegetables because of their low phytochemicals content. Natural fruit and vegetable juices as well as tomato sauces were included in the fruit and vegetable groups because of their high phytochemical content [16, 17, 22].

### Anthropometric assessments

Weight was measured using Omron BF-511 portable digital scales to the nearest 0.1 kg with minimum clothing and in standing position on scale. Height was measured using tape measure on a straight wall to the nearest 0.1 cm, in a standing position without shoes by touching shoulders, buttocks and heels to the wall and head in Frankfurt position. Waist circumference (WC) was measured to the nearest 0.5 cm using non-stretch tape meter, while it is middle of the iliac crown and lowest rib in the standing position. Also, hip circumference was measured from the largest part of the buttocks with an accuracy 0.5 cm. Body mass index (BMI) is calculated by dividing weight (kg) to the square of height (m^2^).

### Physical activity assessment

The short form International Physical Activity Questionnaire (IPAQ) was used to assess frequency and time spent on sedentary, moderate, intensity activities, according to the list of common activities of daily life, over the past week. Activity levels were expressed as Metabolic Equivalent (MET) hours per week [30].

### Diagnosis of metabolic syndrome

MetS was diagnosed according to National Cholesterol Education Program Adult Treatment Panel III (NCEP ATP III). Participants who had at least three of following criteria were diagnosed as participant with MetS: Serum triglycerides ≥150 mg/dL; Serum high-density lipoprotein cholesterol (HDL-C) < 40 mg dL for men and HDL-C < 50 mg/dL for women; Fasting blood glucose ≥100 mg/dL; Blood pressure ≥ 130/85 mmHg and waist circumference ≥ 102 cm for men and > 88 cm for women [31].

### Laboratory measurements

Laboratory measurements included of fasting blood glucose, HDL-C and triglycerides were measured according to a standard laboratory protocol using Pars Azmoon kits (Tehran, Iran) and calibrated Ciba Corning (Switzerland) auto-analysers.

### Assessment of other variables

Blood pressure was measured in a sitting position three times with a 5-min interval between each measurement. Other data including age, gender, marital status, education, job status and history of chronic diseases were collected by trained interviewers.

### Statistical analysis

DPI was categorized based on quartiles ranges. Characteristics of participants were compared throughout quartiles of DPI using one-way analysis for continuous variables and Chi-squared test for non-continuous. To estimate odds ratio and 95% confidence interval (OR (95%CIs)) of MetS and its components in each quartile DPI and overall trend was used binary logistic regressions in crude and multivariable-adjusted models. Analysis stratified by sex were conducted in crude and multivariable-adjusted models. Possible confounders which were considered in models were age (y), sex (men/women) and total energy intake (kcal/day) in the first model. Marital status (married/single/divorce/widowed), physical activity level (sedentary/moderate/active), smoking status (never/former/current), family history of chronic disease (yes/no), educational level (less than high school diploma/college/ university), job status (not employed/employed), house status (owner/not owner), family size (less than 4/more than five), house in square meters (less than 100 square meters/ between 100 to 200 square meters/more than 200 square meters), ethnicity (Native or non-native) in the second model. Along with other variables of the second model, BMI (kg/m^2^) was considered in the third model. Statistical analyses were performed by SPSS statistical software (version 23). *P* values less than 0.05 was statistically considered for significant level.

## Results

General characteristics of the study participants across categories of DPI are presented in Table 1. Mean (SD) of DPI among the first, second, third and fourth quartiles categories was 12.5 (3.15), 19.4 (1.36), 24.53 (1.79) and 36.08 (9.2), respectively. There were no significant differences in BMI, WC, marital status, education, smoking, job status and physical activity levels across quartiles of DPI. The prevalence of MetS and its components was not significantly different across quartiles of DPI except for abdominal obesity (*P* = 0.04).

**Table 1:** General characteristics of the study participants according to quartiles of DPI

Variables	Total ***n*** = 2326	Q1 ***n*** = 581	Q2 ***n*** = 582	Q3 n = 582	Q4 ***n*** = 581	P*
DPI (range)		< 16.7	16.7–21.6	21.7–27.7	> 27.7	
BMI(kg/m^2^)	27.11 ± 4.99	26.88 ± 4.84	26.87 ± 4.96	27.25 ± 5.05	27.43 ± 5.08	0.14
WC(cm)	93.19 ± 13.11	93 ± 13.19	92.5 ± 13.1	93.1 ± 13.3	94.1 ± 12.7	0.20
**Age**; n (%)						0.04
20–29	452(19.6)	128(22.1)	112(19.3)	99(17.2)	113(19.6)	
30–39	494(21.4)	139(24)	119(20.5)	118(20.5)	118(20.5)	
40–49	567(24.5)	121(20.9)	149(25.7)	169(29.3)	128(22.2)	
50–59	457(19.8)	106(18.3)	123(21.2)	103(17.9)	125(21.7)	
60–69	342(14.8)	85(14.7)	77(13.3)	87(15.1)	93(16.1)	
**Sex**; n (%)						0.93
Male	1097(47.3)	270(46.7)	273(47)	281(48.4)	273(47.2)	
Female	1220(52.7)	308(53.3)	308(53)	299(51.6)	305(52.8)	
**Marital status**; n (%)						0.62
Married	2004(86.6)	510(88.1)	494(85.6)	508(87.6)	492(85)	
Single	227(9.8)	48(8.3)	63(10.9)	54(9.3)	62(10.7)	
Divorced or widowed	84(3.6)	21(3.6	20(3.5)	18(3.1)	25(4.3)	
**Physical activity**; n (%)						0.62
Sedentary	688(30.9)	170(30.4)	170(30.4	181(32.1	167(30.6)	
Moderate	809(36.3)	211(37.7)	189(33.8)	210(37.3)	199(36.4)	
Active	730(32.8)	178(31.8)	200(35.8)	172(30.6)	180(33)	
**Education;** n (%)						0.62
Lower than diploma	1234(53.5)	301(52.5)	304(52.7)	324(56.2)	305(52.8)	
High school diploma	700(30.4)	182(31.7)	180(31.2)	164(28.5)	174(30.2)	
**Smoking**; n (%)						0.75
Never and former	2074(91.6)	531(93)	519(91.7)	512(90.1)	512(91.7)	
Current	189(8.4)	40(7)	47(8.3)	56(9.9)	46(8.2)	
**Job status**; n (%)						0.86
Not employed	1373(59.7)	347(60)	338(59)	339(58.9)	349(61)	
Employed	926(40.3)	231(40)	235(41)	237(41.1)	223(39)	
**Chronic diseases family history**; n (%)						0.49
No	504(41.2)	141(44.8)	124(39.4)	124(41.1)	115(39.7)	
Yes	718(58.8)	174(55.2)	191(60.6)	178(58.9)	175(60.3)	
Metabolic syndrome; n (%)	1005(45.2)	227(41)	255(45.5)	253(45.3)	270(49.2)	0.05
Abdominal obesity; n (%)	1025(44.5)	237(41.1)	257(44.4)	249(43.2)	282(49.2)	0.04
Elevated blood pressure; n (%)	559(26.8)	126(22.6)	154(27.4)	157(27.9)	162(29.2)	0.06
Hypertriglyceridemia; n(%)	1423(61.2)	368(63.3)	343(58.9)	349(60)	363(62.5)	0.36
Low serum HDL-C; n (%)	813(35.1)	209(36.2)	207(35.6)	207(35.7)	190(32.9)	0.63
High serum FBS; n (%)	718(30.9)	175(30.1)	178(30.6)	185(31.8)	180(31)	0.93

BMI Body mass index, WC Waist circumference_,_ DPI Dietary phytochemical index, HDL-C High-density lipoprotein cholesterol, FBS Fasting blood sugar, Q quartile, SD Standard deviation

Data are shown mean ± SD for BMI, WC and n(%) for categorical variables. *P*-Value from ANOVA for continious variables and Chi – square test for categorical. *P*-Values < 0.05 was considered significant

### Dietary intake and DPI

Mean dietary intakes of participants across quartiles of DPI are provided in Table 2. Participants in the upper quartile of DPI have higher intakes of whole grain, vegetables, fruits, legumes, nuts, vitamins C, vitamin A, folic acid, pantothenic acid, iron and potassium compare with those in the bottom quartile (*p* < 0.001). Participants in the highest DPI compare to the lowest, have lower intake of energy, total fat, monounsaturated fatty acids (MUFA), saturated fatty acids (SFA), polyunsaturated fatty acids (PUFA), cholesterol, total fiber, vitamin E, zinc, calcium, magnesium (*p* < 0.001). No significant difference was observed across quartiles of DPI for Docosahexaenoic acid (DHA) and Eicosapentaenoic acid (EPA) intake.

**Table 2:** Mean dietary intake of participants by categories of DPI(n = 2326)

	Total n = 2326		Q1 n = 581		Q2 n = 582		Q3 n = 582		Q4 n = 581		P*
Variables	mean	SD	mean	SD	mean	SD	mean	SD	mean	SD	
Dietary PI	23.13	9.95	12.5	3.15	19.4	1.36	24.53	1.79	36.08	9.2	< 0.001
Energy (kca/day)	2863.76	1298.27	3520.42	1440.08	2650.68	1219.47	2495.22	1110.39	2789.73	1157.02	< 0.001
Protein(g/day)	113.8	58.98	133.26	69.13	107.93	57.99	102.37	46.91	111.65	55.18	< 0.001
Carbohydrate(g/day)	402.76	213.28	498.40	277.58	363.92	177.14	345.38	160.65	403.52	184.49	< 0.001
Total Fat(g/day)	111.76	72.15	156.78	93.97	101.80	59.99	92.27	52.12	96.24	54.41	< 0.001
Cholesterol(mg/day)	394.11	382.97	501.18	578.68	385.51	325.27	352.21	261.88	337.64	248.73	< 0.001
SFA (g/day)	31.11	18.12	41.75	23.15	29.47	16.17	26.84	13.60	26.38	13.29	< 0.001
MUFA (g/day)	34.36	24.52	48.61	32.36	31.65	21.08	28.07	17.59	29.13	18.16	< 0.001
PUFA (g/day)	28.54	24.21	36.71	31.02	25.40	20.29	23.20	15.67	28.85	24.98	< 0.001
EPA(mg/day)	0.02	0.08	0.02	0.09	0.02	0.1	0.02	0.07	0.02	0.05	0.742
DHA(mg/day)	0.07	0.21	0.07	0.25	0.07	0.26	0.07	0.19	0.06	0.14	0.686
Total Fiber(g/day)	27.68	24.21	33.70	33.24	22.83	15.83	24.48	22.51	29.71	20.31	< 0.001
Vitamin E (mg/day)	11.12	11.38	12.43	12.92	11.49	11.20	10.52	10.44	10.02	10.67	< 0.001
Vitamin A (RAE/day)	23.38	33.91	19.11	15.64	19.77	27.55	20.07	18.52	34.57	55.62	< 0.001
Vitamin D(μg/day)	1.49	2.40	1.85	3.67	1.46	1.73	1.48	2.33	1.18	1.04	< 0.001
Vitamin K(μg/day)	166.04	243.82	191.19	349.82	160.03	224.99	160.26	175.61	152.70	182.90	< 0.001
Vitamin C(mg/day)	207.27	181.14	164.09	111.82	182.77	135.30	202.85	142.61	279.43	269.46	< 0.001
Folic acid(μg/day)	371.68	211.10	377.25	194.99	336.60	200.11	341.72	169.03	431.26	257.21	< 0.001
VitaminB12(μg/day)	6.03	5.92	7.51	7.01	6.07	5.75	5.42	4.33	5.1302	6.02	< 0.001
Magnesium(mg/day)	335.41	172.25	363.17	176.91	308.04	165.64	307.91	154.04	362.64	182.63	< 0.001
Potassium(mg/day)	4072.06	2098.04	4197.37	1950.44	3652.56	1823.79	3810.11	1869.87	4629.36	2536.55	< 0.001
Zinc (mg/day)	11.90	5.93	13.66	7.05	11.17	5.63	10.77	4.71	11.98	5.67	< 0.001
Fe(mg/day)	42.33	56.92	38.28	46.98	38.99	44.58	39.77	44.42	52.29	81.72	< 0.001
Fruits(g/day)	585.45	512.03	386.67	264.58	495.06	356.73	604.30	418.80	855.89	745.9	< 0.001
Vegetables (g/day)	216.26	246.33	198. 7	235.6	196.85	158.86	215.69	297.83	253.82	267.4	< 0.001
Legumes (g/day)	47.25	58.01	45.42	59.78	40.95	38.98	44.11	36.19	58.55	83.09	< 0.001
Nuts (g/day)	23.01	34.01	25.84	34.20	17.21	20.59	18.02	22.16	31.01	49.18	< 0.001
Whole grains (g/day)	73.55	68.49	49.41	53.29	68.66	51.01	77.01	59.27	99.11	92.54	< 0.001
Fructose (g/day)	30.67	28.36	36.15	41.40	26.82	20.24	26.78	18.95	32.94	25.98	< 0.001

DHA Docosahexaenoic acid, MUFA Monounsaturated fatty acid, SFA Saturated fatty acid, PUFA Polyunsaturated fatty acids, EPA Eicosapentaenoic acid

DPI Dietary phytochemical index, Data are shown mean ± SD, ^*^Pvalues from ANOVA and were considered < 0.05 significant

### DPI and MetS and its components

The odds ratio and (95% CIs) of MetS and its components across quartiles of DPI are presented in Table 3. After adjustment for age, sex and energy intake, the risk of MetS (OR: 0.72, 95% CI = 0.55–0.93, *P* = 0.01) and abdominal obesity (OR: 0.70, 95% CI = 0.53–0.93, P = 0.01) decreased significantly in the second quartiles of DPI compared to the first. Also odds of abdominal obesity in the fourth quartiles of DPI compared to the first, was significantly decreased (OR: 0.75, 95% CI = 0.58–0.99, *P* = 0.04). In addition to the risk of MetS (OR: 0.58, 95% CI = 0.38–0.88, *P* = 0.01) and elevated blood pressure (OR: 0.59, 95% CI = 0.38–0.92, *P* = 0.02) decreased in the second quartile of DPI compared to the first after further adjustments for marriage status, physical activity, education level, Smoking, job status, house status, family size, house in meters, ethnicity, hypercholesterolemia, chronic disease family history. In the third model, the risk of MetS (OR: 0.63, 95% CI = 0.41–0.96, *P* = 0.03) and elevated blood pressure (OR: 0.62, 95% CI = 0.40–0.96, P = 0.03) significantly decreased in the second quartile of DPI compared to the first in full adjustmens. There was not any significant relationship between DPI and other components of MetS in crud and full adjustments model (data are not shown).

**Table 3:** OR(95% CI) for metabolic syndrome and its components according to quartiles of DPI

Variables	Q1 (n = 581)	Q2 (n = 582)	Q3 (n = 582)	Q4 (n = 581)	P trend
**Metabolic syndrome**					
Crude	1(ref.)	0.71 (0.56–0.91) *P* = 0.006	0.86(0.68–1.09) *P* = 0.22	0.85(0.67–1.08) *P* = 0.20	0.01
Model 1^a^	1(ref.)	0.72 (0.55–0.93) P = 0.01	0.88 (0.68–1.13) *P* = 0.32	0.88(0.68–1.13) *P* = 0.33	0.02
Model 2^b^	1(ref.)	0.58 (0.38–0.88) *P* = 0.01	0.90 (0.6–1.33) *P* = 0.59	0.79(0.53–1.18) *P* = 0.26	0.02
Model 3^c^	1(ref.)	0.63 (0.41–0.96) P = 0.03	0.92 (0.61–1.39) *P* = 0.72	0.78(0.51–1.17) *P* = 0.23	0.08
**Abdominal obesity**					
Crude	1(ref.)	0.72(0.57–0.91) P = 0.006	0.82(0.65–1.03) *P* = 0.10	0.78(0.62–0.98) *P* = 0.03	0.01
Model 1^a^	1(ref.)	0.70(0.53–0.93) P = 0.01	0.81(0.62–1.07) *P* = 0.14	0.75(0.58–0.99) P = 0.04	0.03
Model 2^b^	1(ref.)	0.66(0.42–1.02) *P* = 0.06	0.82(0.54–1.25) *P* = 0.36	0.83(0.54–1.26) *P* = 0.38	0.08
Model 3^c^	1(ref.)	0.74(0.43–1.27) *P* = 0.28	0.86(0.51–1.44) *P* = 0.58	0.73(0.43–1.23) *P* = 0.24	0.41
**Elevated blood pressure**					
Crude	1(ref.)	1.28(0.98–1.69) P = 0.06	1.32(1.01–1.73) P = 0.04	1.41(1.07–1.85) P = 0.01	0.01
Model 1^a^	1(ref.)	1.28(0.95–1.72) *P* = 0.09	1.32(0.98–1.77) P = 0.06	1.35(1.01–1.81) P = 0.03	0.05
Model 2^b^	1(ref.)	0.59(0.38–0.92) P = 0.02	0.81(0.53–1.22) *P* = 0.31	0.96(0.64–1.45) *P* = 0.87	0.01
Model 3^c^	1(ref.)	0.62(0.40–0.96) P = 0.03	0.83(0.55–1.26) *P* = 0.39	0.96(0.63–1.45) *P* = 0.84	0.03

OR Odds ratio, CI Confidence interval, PI phytochemical index, Q quartile

^a^ Adjusted for age, sex and energy intake, ^b^ Additional adjustment for marriage status, physical activity, education level, smoking, job status, house status, family size, house in meters, ethnicity, hypercholesterolemia, chronic disease family history. ^c^ Additional adjustment for BMI

### DPI and MetS its components stratified by gender

Odds ratio (95% CIs) of MetS across different categories of DPI stratified by sex are shown in Table 4**.** In women, after adjustment for confounders, risk of MetS significantly decreased in the highest quartile of DPI as compared to the lowest (OR: 0.41, 95% CI = 0.22–0.76, *P* = 0.005). There was not any significant relationship between DPI and MetS components stratified by sex (data are not shown).

**Table 4:** OR (95% CI) for metabolic syndrome by quartiles of DPI stratified by gender (*n* = 2326)

Sex	Q1	Q2	Q3	Q4	P_**trend**_
**Men**					
Crude	1(ref.)	0.72(0.49–1.05) P = 0.09	1.04(0.72–1.49) *P* = 0.83	1.03(0.72–1.48) P = 0.83	0.12
Model 3^*^	1(ref.)	0.54(0.27–1.07) *P* = 0.08	1.39(0.77–2.51) *P* = 0.27	1.36(0.74–2.40) P = 0.31	0.15
**Women**					
Crude	1(ref.)	0.66(0.47–0.92) P = 0.01	0.72(0.51–1) *P* = 0.05	0.73(0.52–1.02) P = 0.06	0.02
Model 3^*^	1(ref.)	0.74(0.40–1.38) *P* = 0.35	0.62(0.34–1.14) *P* = 0.12	0.41(0.22–0.76) P = 0.005	0.70

OR Odds ratio, CI Confidence interval, DPI Dietary phytochemical index, Q quartile

^*^ Adjusted for age, energy intake, marriage status, physical activity, education level, smoking, job status, house status, family size, house in meters, ethnicity, hypercholesterolemia, chronic disease family history, BMI

## Discussion

The findings of the present study indicated a reduction risk of high blood pressure and MetS with higher adherence to DPI after adjusting a wide range of possible confounder variables. Decrease in the risk of abdominal obesity was associated with higher DPI scores, independent of age, sex and total energy intake. Women with most conformity of DPI showed lower odds of MetS. These findings remained significant after a full adjustment for confounders. Due to the insufficient evidences on the relationship between DPI and MetS or its components, the results of this study shine new insights about the relationship between DPI with odds of MetS and its components in a large sample of Iranians adults as a representative of the Middle Eastern countries.

Our results also indicated that a diet with higher DPI was associated with a diminished risk of high blood pressure and MetS. These findings are in agreement with the results of former studies, which reported that higher amounts of vegetables and fruits [32, 33], whole grains [34, 35], nuts [36, 37], legumes [38], are inversely associated with hypertension. Other studies have shown that the consumption of phytochemical abundant foods may prevent hypertension and MetS [39–41]. These findings may be explained by the synergistic effects of phytochemicals together with its antioxidant and anti-inflammatory properties, as well as high intake of antioxidant vitamins including vitamin C and vitamin A that can inverse insulin resistance [33, 40, 42]. Further evidences suggest the protective effect of a higher intake of potassium and folate on endothelial function, MetS and hypertension [33, 43]. In contrast, a cross-sectional study on Iranian adults observed no significant association between DPI and the odds of hypertension and MetS [44]. Such contradictions may be due to the differences in sampling size and dietary habits.

Our results also showed a significant reverse relationship between abdominal obesity and higher conformity of DPI, which are consistent with a longitudinal study on adults, reporting that an increase in energy intake from phytochemicals-rich foods precludes weight gain and adiposity [45]. A cross-sectional investigation on 54 adults aged between 18 and 30 years showed that DPI score was inversely related to WC (25). The protective role of phytochemicals against WC may be mediated by inhibitory role of some polyphenols in proliferation of pre-adipocytes, reducing adipogenesis, and stimulating lipolysis [46, 47]. However, our findings did not remain significant after further adjustment for confounders, which may be due to the differences in general and genetic characteristics of the subjects.

In the present study, the analysis stratified by sex showed that odds MetS reduced in women with highest compliance of DPI. In line with our findings, a cross-sectional study showed a lower risk of the MetS among women with higher intakes of fruits and vegetables [48]. Another study found that moderate and high intake of fruits could alleviate MetS in women [49]. Some studies have also reported that phytochemicals in food sources could improve lipid metabolism in middle-aged women especially with menopause [50, 51]. Several possible mechanisms of higher adherence of DPI and MetS in women can be described. For example, women with higher intake of DPI have lower levels of C-reactive protein, oxidative stress and inflammation than men due to an interaction between sex hormones and some phytochemicals intake such as isoflavones with similar structure to estrogen [52–56].

The advantages and limitations of the present study can be summarized as follows. Population-based design was the most important strength of this study. Face-to-face interviews in a large sample of population using trained interviewers were other strengths. Dietary intakes were assessed with a validated questionnaire. Nevertheless, the major limitation of the cross-sectional design of this study was rooted in the inability to determine the direction of relations. The possibility of not considering all possible confounders was another one. Also, the dietary phytochemicals quality of the participants in the same DPI was not determined in term of the variation in intake food containing phytochemicals.

## Conclusions

More adherence to DPI is probably related to reduced risk of MetS **especially in women**. Interventional studies are needed to discover causal relations and relevant underlying mechanisms.

## Data Availability

The YaHS database is closed. The data was provided by the CI of the study, professor Masoud Mirzaei in line with the study protocol. http://www.yahs-ziba.com

## Abbreviations

DPI: Dietary Phytochemical Index
MetS: Metabolic Syndrome
YaHS: Yazd Health Study
TAMYZ: Taghzieh Mardom-e-YaZd
YNS: Yazd Nutrition Survey
FFQ: Food Frequency Questionnaire
WC: Waist Circumference
IPAQ: International Physical Activity Questionnaire
MUFA: Monounsaturated Fatty Acids
SFA: Saturated Fatty Acids
PUFA: Polyunsaturated Fatty Acids
HDL-C: Serum High-Density Lipoprotein Cholesterol
FBS: Fasting Blood Glucose
DHA: Docosahexaenoic Acid
EPA: Eicosapentaenoic Acid
BMI: Body Mass Index
MET: Metabolic Equivalent
SD: Standard Deviation
